# Four Amino Acids within a Tandem QxVx Repeat in a Predicted Extended α-Helix of the Smad-Binding Domain of Sip1 Are Necessary for Binding to Activated Smad Proteins

**DOI:** 10.1371/journal.pone.0076733

**Published:** 2013-10-11

**Authors:** Andrea Conidi, Veronique van den Berghe, Kris Leslie, Agata Stryjewska, Hua Xue, Ye-Guang Chen, Eve Seuntjens, Danny Huylebroeck

**Affiliations:** 1 Laboratory of Molecular Biology (Celgen), Department of Development and Regeneration, Katholieke Universiteit Leuven, Leuven, Belgium; 2 Department of Cell Biology, Erasmus University Medical Center, Rotterdam, The Netherlands; 3 The State Key Laboratory of Biomembrane and Membrane Biotechnology, Tsinghua-Beijing Centre for Life Sciences, School of Life Sciences, Tsinghua University, Beijing, China; National Cancer Center, Japan

## Abstract

The zinc finger transcription factor Smad-interacting protein-1 (Sip1; Zeb2, Zfhx1b) plays an important role during vertebrate embryogenesis in various tissues and differentiating cell types, and during tumorigenesis. Previous biochemical analysis suggests that interactions with several partner proteins, including TGFβ family receptor-activated Smads, regulate the activities of Sip1 in the nucleus both as a DNA-binding transcriptional repressor and activator. Using a peptide aptamer approach we mapped in Sip1 its Smad-binding domain (SBD), initially defined as a segment of 51 amino acids, to a shorter stretch of 14 amino acids within this SBD. Modelling suggests that this short SBD stretch is part of an extended α-helix that may fit the binding to a hydrophobic corridor within the MH2 domain of activated Smads. Four amino acids (two polar Q residues and two non-polar V residues) that form the tandem repeat (QxVx)_2_ in this 14-residue stretch were found to be crucial for binding to both TGFβ/Nodal/Activin-Smads and BMP-Smads. A full-length Sip1 with collective mutation of these Q and V residues (to A) no longer binds to Smads, while it retains its binding activity to its cognate bipartite target DNA sequence. This missense mutant Sip1(AxAx)_2_ provides a new molecular tool to identify SBD (in)dependent target genes in Sip1-controlled TGFβ and/or BMP (de)regulated cellular, developmental and pathological processes.

## Introduction

The Transforming Growth Factor type β (TGFβ) system controls many cellular processes including proliferation versus differentiation, (de)adhesion, epithelial-mesenchymal transition (EMT), and cell migration and accompanying cell shape changes [1]–[4]. TGFβ family signaling, including via Nodal and Bone Morphogenetic Proteins (BMPs), is also crucial during embryogenesis in induction, patterning and morphogenesis, and for the regulation of stem/progenitor cells and their niche in the embryo and the adult animal [5], [6]. In each of these normal processes, the signaling by this ligand-receptor system together with its intracellular signal transduction involving Smad proteins and protein-kinase based non-Smad signaling, is under tight control at multiple levels and by various mechanisms [7]–[10]. The Smads engage in interactions with a wide variety of proteins outside and inside the nucleus. Many of the nuclear partners for receptor-activated Smads are important transcription factors or co-factors that steer cell fate determination and cellular behaviour [6], [11]. Understanding how each of these many Smad-interacting proteins (SIPs) precisely function when bound to Smads, and whether they can also function Smad-independently, remains both in the Smad and SIP fields a relevant challenge.

Smad-interacting protein-1 (Sip1, also named Zeb2 and Zfhx1b) was one of the first identified SIPs [12]. In most assays involving its expression from transfected vectors, full-length Sip1 represses the transcription of endogenous candidate target genes or target gene promoter based reporters. Based on candidate target promoter analysis and *in vitro* DNA-binding, full-length Sip1 binds to DNA with two zinc fingers, present in each of its two zinc finger rich clusters, to a separated repeat of mainly CACCT(G) in gene regulatory regions [13]. In this way Sip1 down regulates *E-cadherin (Cdh1)* mRNA levels [14], [15]. *Cdh1* is also regulated by many other factors and mechanisms during EMT (*e.g.* Snail family zinc finger repressors, Rho and micro-RNAs; for a review, see [16]) [17], [18] and high Sip1 levels in several Cdh1-low/null epithelial-derived tumors are indicative for bad prognosis [19]–[21]. Mouse Sip1 is a 1215 amino acid (aa)-long protein (1214 in human) containing several functional domains. Binding of Sip1 to both the TGFβ/Nodal/Activin-Smads (Smad2 and 3) and BMP-Smads (Smad1, 5 and 8) in ligand-stimulated cells depends on its 51 aa-long Smad-binding domain (SBD) located in-between the two zinc finger clusters [12]. In addition, a short segment located in the extreme N-terminal part of Sip1 serves binding to the chromatin remodelling complex NuRD. This NuRD-interaction motif (NIM) of Sip1 is mutated - and no longer binds NuRD - in a patient with mild symptoms of Mowat-Wilson syndrome (MWS) [22]. It is unknown whether the SBD and NIM of Sip1 function independently of each other or not.

Mutations in the SIP1-encoding gene *ZFHX1B* cause MWS (MIM #235730), which is characterized by severe intellectual disability, typical craniofacial malformation and in most patients epilepsy and Hirschsprung disease [23]–[25]. Studies with various *Sip1* knockout mice show that the Hirschsprung disease and the craniofacial malformations have their origin in defects in neural crest cells. Additional studies in mutant mice also pointed at defects in sensory neurogenesis, in particular in dorsal root ganglia, and some MWS patients are indeed less sensitive to pain [26]–[29]. Sip1 also regulates early neural differentiation *in vivo* and in cultured embryonic stem cells [30]–[35], and is later an important factor in the timing of neurogenesis and gliogenesis in the cortex of the embryonic mouse brain [36]. Specific loss of Sip1 in GABAergic interneurons affects their fate and disrupts their guided migration from the ventral telencephalon to the cortex in the mouse brain [37], [38]. During myelinogenesis, Sip1 exerts anti-BMP activity and thereby promotes myelination [39]. It achieves this by binding to activated BMP-Smads that normally induce a set of BMP-induced genes encoding inhibitors of myelinogenesis, leading to Sip1/Smad-mediated repression of these same genes. Sip1 can also activate transcription (for a discussion, see [40]). For example, during myelinogenesis Sip1 directly activates transcription of *Smad7*, which provides additional negative control of inhibitory BMP signaling during myelinogenesis [39]. These observations raise important questions about Sip1 and SIP transcription factors in general: do they exert an anti-BMP activity also within other cell types (in culture and *in* *vivo*)? Does their activity always occur in concert with Smads? If not, what is then the subset of SIP target genes that depends on Smad-SIP interaction?

Peptide aptamers are recombinant proteins obtained through in-frame insertion of short peptides in an appropriately selected scaffold protein. They are good tools to document protein-protein interaction via specific domains or, when expressed in cells at sufficiently high levels, to interfere with protein function mainly by sequestering their cognate targets [41]. Aptamers also provide possibilities to screen for agents (drugs, synthetic compounds) that mimic the structure of the identified peptides and that can be tested for their therapeutic potential [42], [43]. Thioredoxin (TrxA) of *E. coli* is frequently used as scaffold because a loop encompassing its active site is ideally suited for displaying conformationally constrained peptides [41], [43]. Such TrxA-based aptamers have meanwhile been used to identify structural elements or important regions of other proteins and SIPs, including FoxH1, CBP, Lef1 and SARA [44], [45].

Using a combination of peptide aptamers and structural bioinformatics, we identified a specific 14 aa-long linear sequence within the SBD of Sip1 that represents the minimal binding segment for Smads. Mutagenesis of 4 amino acids only, within this stretch, in full-length Sip1 abolished Sip1 binding to Smads. Such new mutant Sip1 will facilitate the identification of Sip1’s Smad-(in)dependent actions and target genes in future functional studies.

## Materials and Methods

### Cell Culture and Plasmid Transfection

Human embryonic kidney (HEK) 293T cells were cultured in Dulbecco’s Modified Eagle Medium (DMEM) supplemented with 4.5 g/l glucose, 10% (v/v) foetal bovine serum and 1% (v/v) L-Glu (both from Gibco, Life Technologies). NMe cells were grown as described in [46]. Plasmids were transfected using Lipofectamine-2000 (Life Technologies) according to the manufacturer’s instructions.

### Plasmids and Cloning

**Figure 1:**
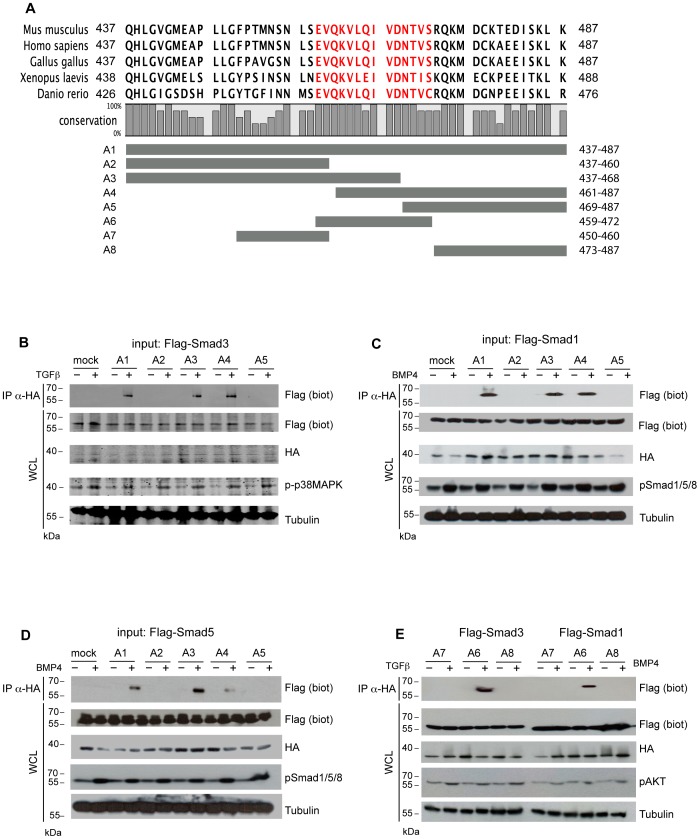
TGFβ and BMP activated Smads bind to the same aa459–472 segment of (mouse) Sip1. A) Sip1 SBD sequence conservation in five different vertebrates, and schematic representation of the peptide aptamers used in this study. The numbering shown for the aptamers at the right applies to the mouse protein sequence; the histograms reflect strong versus weaker conservation amongst the animal species. The aptamer A1 represents the insert in the Trx protein scaffold of the entire 51 aa-long SBD, as defined previously [12]; the aptamers A2–A8 are C- and/or N- terminal truncations of A1. B–D) Co-IP experiments in extracts from transfected HEK293T cells show that both TGFβ (B) and BMP (C,D) activated (Flag-tagged) Smads bind only to the aptamers A1, A3 and A4, indicating that the shared region shared between A3 and A4 is responsible for the interaction. E) Flag-tagged Smad3 and Smad1 proteins were specifically bound by a 14 aa-long sequence represented by the A6 aptamer (sequence in red in panel A), and comprehending the tandem repeat (QxVx)_2_ (for details, see main text). The activation of the TGFβ family pathway was assessed using p-p38MAPK or pAKT levels; Tubulin detection was used as loading control. Panel B was acquired using a Digital Chemiluminescence System (Bio-Rad; see Materials and Methods).

For expression of the Trx-SBD aptamers in mammalian cells, we inserted the cDNA encoding the entire mouse Sip1 SBD (named A1; Figure 1A) or fragments thereof (A2 to A5), amplified by PCR using primers carrying 5′-*Rsr*II sites, into the unique *Rsr*II site of pCI-NLS-HA-Trx (kindly provided by F. M. Hoffmann, Madison, USA; see [44]). The sequences encoding the aptamers A6, A7 and A8 were obtained by single-strand oligonucleotide annealing and inserted into pCI-NLS-HA-Trx as described above. The cloning of full-length, wild-type (WT) Myc-tagged Sip1 in expression vectors has been described [15]. The full-length Sip1(AxAx)_2_ mutant cDNA was obtained by PCR using complementary primers carrying the desired mutations together with primers respectively covering the ATG initiation codon and the stop codon of mouse Sip1 cDNA. This mutant cDNA was inserted in pCS3-myc (adding an in-frame N-terminal c-Myc tag to Sip1) and then used for transfection of HEK293T or NMe cells. The mutant cDNA was also inserted between the *Cla*I and *Not*I sites in pCIG (pCAGGS-IRES-eGFP; see [38]) for focal electroporation experiments in embryonic brain slices. In the latter approach, the eGFP coding region was lost with the cloning. As control a pCIG-Sip1WT encoding vector was used. The Sip1ZnF mutant no longer binds to DNA [13]. Briefly, it is mutated in essential residues of 2 zinc fingers of the N-terminal zinc finger cluster and of 2 zinc fingers of the C-terminal zinc finger cluster of Sip1. To visualize electroporated cells in the brain slices the plasmid pCALNL (Addgene) was used.

### DNA-IP

Complementary sense and anti-sense oligodeoxyribonucleotides containing the Sip1-binding E-boxes of the Activin-inducible *Xbra2* gene [12], [13] were resuspended in 1× annealing buffer (50 mM Tris-HCl pH7.8, 10 mM MgCl_2_) to a final concentration of 400 ng/ µl and incubated for 2 min at 95°C, followed by 15 min at 70°C, 15 min at 37°C, 10 min at 25°C and, finally, 15 min at 4°C. Annealing was confirmed by electrophoresis in 2% agarose gel. The annealed oligonucleotides were then incubated with pre-cleared Streptavidin conjugated magnetic beads (Dynabeads M280, Life Technologies) for 1 hour at 4°C on an orbital shaker at low speed. Nuclear versus cytoplasmic fractionation was performed on transfected HEK293T cells using the Ne-Per kit (Pierce) following the manufacturer’s protocol with the modification that the nuclear lysate was homogenized by passing 5 times the protein extract through a 22-G needle every 10 minutes. 15 µg of nuclear lysate were subjected, in parallel with the cytoplasmic fraction, to SDS-polyacrylamide gel electrophoresis to verify the presence of Sip1 or Sip1 mutant proteins in these cell fractions. Lamin-C was used as nuclear marker protein and loading control. The prepared nuclear extracts were added to the beads-oligonucleotides and incubated for 16 hours at 4°C with gentle shaking. They were then washed with PBS containing 0.2% BSA, and the bound proteins were eluted from the beads with 5×Laemmli loading buffer at 95°C for 10 min, and afterwards separated by electrophoresis in 6% SDS-polyacrylamide gels.

### Co-immunoprecipitation, Western Blotting and Antibodies

Transfected HEK293T cells were stimulated 24 hours after transfection with recombinant human (rh) TGFβ1 (5 ng/ml; R&D Systems) or rhBMP4 (25 ng/ml; R&D Systems). After 1.5 hour, the cells were lysed using lysis buffer (170 mM NaCl, 10 mM EDTA, 50 mM Tris-HCl pH7.4, 0.5% NP-40, supplemented with proteases inhibitors (Roche) and phosphatase inhibitors (1 mM NaF, 10 mM NaVO_3_)). 500 µg of protein from these extracts were incubated overnight with magnetic G-protein-coupled beads (Dynabeads) bound to anti-HA or anti-FLAG antibody. Elution of the captured proteins from these beads was done using 5×Laemmli sample buffer and heating at 95°C for 10 min. Protein lysates (15 µg in total for the detection of Flag-Smads, phosphoAKT (pAKT) and Tubulin; 60 µg in total for detection of HA-tagged aptamers) were separated by SDS-PAGE and then transferred to a PVDF Membrane (GE Healthcare). The blots were then incubated with skimmed non-fat dry milk (5% w/v) in TBS containing 0.25% Tween–20 for 1 hour and then incubated for 16 hours at 4°C with one of the following antibodies (at the indicated dilutions): anti-phosphoSmad5 (detecting pSer463/pSer465 of Smad1 as well) (Epitomics/AbCam; 1∶1000), anti-phosphoAKT (Cell Signaling; 1∶1000), anti-phospho p38MAPK (Cell Signaling; 1∶1000), anti-myc (clone 9E10, Santa Cruz; 1∶1000), anti-FLAG M2 and biotinylated anti-FLAG M2 (Sigma; 1∶2500), anti-HA (CoVance; 1∶1000), anti-tubulin (ProBio; 1∶3000), anti-Lamin C (Novocastra; 1∶1000). We tried several antibodies for detection of phosphoSmad3 (pSmad3) levels, unfortunately none of the commercially available antibodies that we tested was good enough to verify accurately the status of Smad3 activation in Western Blots. Therefore, we decided to use pAKT and p-p38MAPK as an alternative marker for TGFβ pathway activation. After three washes of 15 min each, the blots were incubated with the appropriate Horse Radish Peroxidase (HRP)-conjugated secondary antibody (1∶25000, Jackson ImmunoResearch). For detection of the biotinylated Flag antibody a Streptavidin HRP-conjugated secondary antibody was used (Perkin Elmer). Signals were visualized using ECL reagent (Amersham) and developed using Fuji X films (Fuji) or Digital Chemiluminescence System (Bio-Rad).

### Luciferase Assay

2.5×10^4^ HEK293T cells were seeded per well of a 96-well plate and transfected with pGL3-Cdh1 plasmids containing the promoter region (−308/+21, where the transcription start site is +1) of human *Cdh1* [14], and one of the Sip1-encoding vectors and/or a vector encoding a constitutively active (c.a., *i.e.* ligand-independent) variant of the Smad2/3-activating Alk4 receptor (c.a.Alk4). The cells were lysed by repeated freeze/thawing in Tropix lysis buffer (100 mM KH_2_PO_4_ pH7.8, 0.2% Triton X-100, 1 mM DTT) 24 hours after transfection. For luciferase quantification the Luciferase Assay Substrate (from Promega) was used. Normalization was to β-galactosidase activity produced from a co-transfected pCMV-βGal vector (Clontech) and assayed using Galscreen Tropix (Applied Biosystems). Typically, three independent experiments were performed in quadruplicate. Absolute values were standardized to the (respective) controls (mock = 1).

### 3D-modelling

The mouse Sip1 sequence aa437–487, encompassing the initially defined 51 aa-long SBD [12], was submitted to the Phyre2 server and several pdb files were obtained [47]. Each file with the different predicted structure was then analysed using PyMol software (PyMOL Molecular Graphics System, Version 1.5.0.4 Schrödinger, LLC). Next, we subjected the Sip1 sequence aa417–503 to a One-to-One threading modelling using as template structure the d1vpra1.pdb file. Volume occupancy was visualized using PyMol software, while electrostatic potential analysis and RMSD calculations were performed with VMD software [48], [49].

### Stress Fiber Quantification in Cultured Cells

12.5×10^4^ NMe cells were seeded in each well of a 24-well plate coated with PBS −0.1% gelatine, and then transfected in suspension with ON-TARGET plus SmartPOOL siRNA that targets mouse Sip1 (25 nM final concentration; L-059671-00-0005, Dharmacon) or a control Non-Targeting siRNA (siCTRL, Dharmacon) and the plasmid of interest at a final concentration of 1.2 µg/ml. 16 hours after transfection, the cells were stimulated with 5 ng/ml of TGFβ1 for 48 hours, adding the same concentration of fresh TGFβ1 upon medium replacement after 24 hours. Cells were then fixed in PBS containing 2% paraformaldehyde for 10 min on ice, quenched in 50 mM NH_4_Cl two times for 5 min, and then incubated with Blocking Reagent (Roche) in PBS −0.2% Triton X-100 (PBST) for 1 hour. After two washes of 5 min each with PBST, the slides were incubated with anti-Sip1 antiserum (GeneScript; 1∶100) or anti-Myc antibody (1∶500) in Blocking Reagent in PBST for 1.5 hour. After three washes with PBST, the cells were incubated for 1 hour with anti-phalloidin (488-conjugated, 1∶5000; Alexa Fluor) and a secondary anti-mouse Cy3 conjugated antibody (Alexa Fluor). The cells were then stained with phalloidin to detect the formation of stress fibers. At least 4 different and independent regions of each slide were analysed by microscopy. The area covered by stress fibers (green staining; number of pixels/µm^2^) was quantified using ImageJ software. The acquired images were converted to 8-bit grayscale and the foreground/background colors were inverted. The threshold was adjusted until the phalloidin fluorescence was excluded by the threshold limits. Stained areas were measured in pixels setting the scale of the image in µm. Statistics was performed using the Student T-test.

### Animals – Ethics Statement

We confirm that all animal experiments were done according to the latest regulations set by the Belgian authorities that follow the European Parliament and Council Directive 2010/63/EU on the protection of animals used for scientific purposes. This is certified by authorization to our laboratory via certificate LA1210584 from the Belgian Ministry of Public Health, Safety of the Food Chain and Environment, including from its inspection department and its deontological committee (file 11/2010; August 4, 2011). We confirm we also obtained approval by the Animal Care Committee of KU Leuven, the acting Institutional Animal Care and Use Committee (IACUC) of KU Leuven. Mouse lines were maintained in a CD1/Swiss background. Homozygous *Sip1 (exon7)* “floxed” mice [50] were crossed with homozygous *RCE^fl/fl^* (which stands for *ROSA26R^CAG-loxP-stop-loxP-EGFP^*; [51]) reporter mice. *Sip1^fl/fl^;RCE^fl/fl^* mice were mated with Nkx2-1-*Cre* mice [52] that were heterozygous for *Sip1* to obtain *Cre;Sip1^fl/KO^* (KO = knockout) mutant embryos (referred to as Sip1;RCE|Nkx2-1).

### Focal Electroporation in Embryonic Brain Slices

Focal electroporation was done as described previously [38]. E13.5 brains from Sip1;RCE|Nkx2-1 embryos were dissected in ice-cold Hepes-buffered Leibovitz’ L15-medium supplemented with glucose, and then embedded in 4% low-melting point agarose. Coronal slices (300 µm) were sectioned using a vibratome (HM650V, Microm). Injection in the MGE was performed with the aforementioned plasmids (0.5 µg/ µl) and 4% Fast Green (Sigma-Aldrich). The Cre-dependent dsRed CALNL plasmid (1 µg/ µl) was used as control or co-electroporated to label the neurons in which Sip1 was deleted by Cre. Injected slices were electroporated using a cover square platinum plate electrode (CUY701P20L) and a petridish square platinum plate electrode (CUY701P20E) (both from Sonidel) via a BTX electroporator (ECM830, Harvard Apparatus) (5 pulses of 150 V and 5 ms duration with 100 ms intervals). The electroporated slices were transferred to poly-L-Lysin (Sigma-Aldrich) and Laminin (Sigma-Aldrich) coated inserts (Millicell Cell Culture insert, pore size 0,4 µm, Millipore) and cultured for 3 days *in vitro* (DIV) using an air-interface protocol [53]. Slices were fixed with 4% paraformaldehyde, mounted on slides and analyzed via confocal microscopy (Nikon A1R Eclipse Ti). For each condition, we quantified the total amount of RFP-positive (RFP+) cells in the slice and calculated the proportion of RFP+ neurons that reached the cortex using ImageJ software. Statistical significance of the difference between each condition was calculated using the Chi-square test.

## Results

### Peptide Aptamers Identify a Stretch of 14 Amino Acids within the 51 Amino Acids-long SMAD-binding Domain (SBD) Necessary to Confer Binding of Sip1 to Smads

The initially mapped SBD of Sip1 was a 51 aa-long linear sequence encompassing aa437–487 of mouse Sip1. This SBD is necessary and sufficient to interact with the MH2 *i.e.* C-terminal domain of Smad1 in GST-based pull-down assays, with the MH2 domain of Smad2 and 3 and with the MH2 domain of Smad1, 5 and 8 in yeast two-hybrid assay, and in BMP pathway-activated mammalian cells [12,31; Dzwonek, Conidi, van Grunsven, Huylebroeck and Verschueren, *unpublished results*). We wanted first to locate the minimal segment in this SBD needed for binding to activated Smads. Truncated SBD peptides (numbered A1–A5, see Figure 1A) were inserted at the cDNA level in HA-tagged Trx in a transient expression vector for mammalian cells [44]. Next, we tested by co-immunoprecipitation (co-IP) whether these SBD-Trx proteins were able to bind to Smads. For this, HA-tagged aptamer and FLAG-tagged Smad encoding constructs were transfected to HEK293T cells, stimulated with TGFβ1 or BMP4, as well as unstimulated cells. Only the A1 (the 51 aa-long SBD), A3 (lacking the last 19 aa of these 51 aa) and A4 (lacking the first 24 aa of SBD) SBD-Trx polypeptides bound to Smad3 in conditions of activation, as documented by the levels of phosphorylated p-p38MAPK (Figure 1B). These results, obtained by using Sip1 SBD-based aptamers, confirm our previous observations with full-length, N-terminally tagged Sip1 (Dzwonek, Conidi, Verschueren and Huylebroeck, *unpublished results*) that Sip1 binds to activated Smads only. Interestingly, the BMP-activated Smads tested here, *i.e.* Smad1 and Smad5 (Figure 1C,D), were bound by these same three aptamers. This indicates that the region shared between A3 and A4, *i.e.* 14 aa (aa459–472), is necessary for the interaction of Sip1 with Smads in ligand-stimulated cells.

To narrow down further the sequence necessary for Smad binding, we designed additional aptamers covering segments of the A3/A4 sequence, *i.e.* A6, which precisely co-incides with the segment 459–472; A7, a more N-terminally located segment in A3, but absent from A4; and A8, a C-terminal segment in A4, but absent from A3 (see Figure 1A). A new round of co-IP experiments showed that only A6 was able to bind to activated Smads (see Figure 1E for Smad3 and Smad1; pAKT levels were used here to verify TGFβ or BMP4 pathways activation. We therefore conclude that aa459–472 (*i.e.* the sequence of A6; see Figure 1A) within mouse Sip1 is the minimal linear SBD sequence tested here that is able to mediate interaction with activated Smads.

### A Predicted, Structurally Conserved Element of the Sip1 Smad-binding Domain is Necessary for the Interaction with the MH2 Domain of Activated Smads

**Figure 2:**
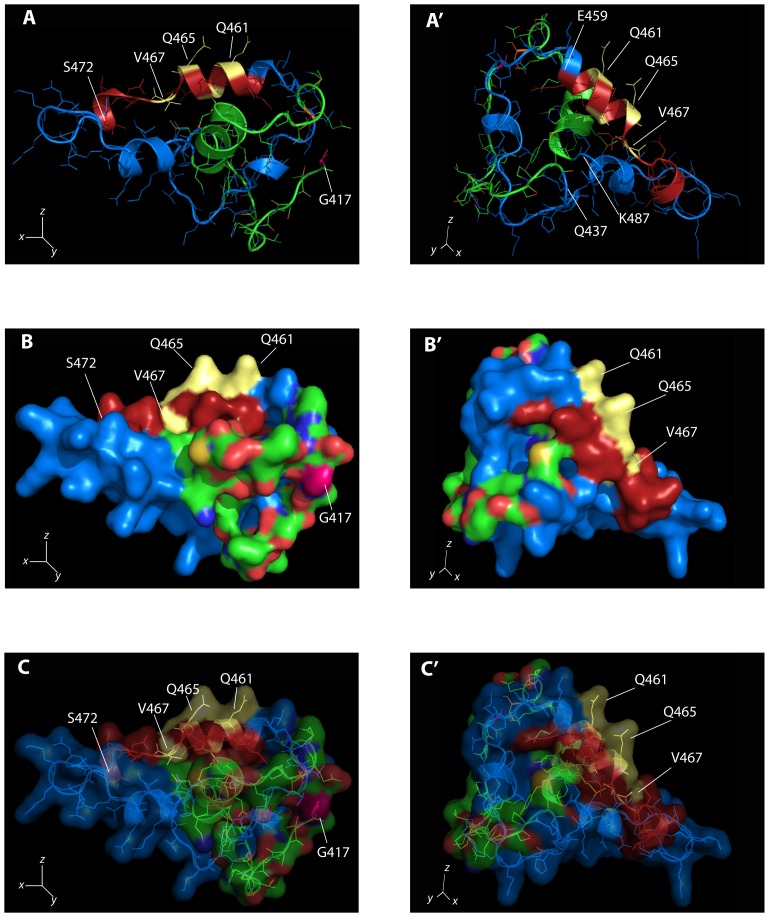
3D modelling of the Sip1 SBD sequence and structural features of the 459–472 sequence. Two orientations of the same model are depicted. Panels A and A′ represent the “cartoon model” highlighting the structural elements present in the SBD of WT Sip1. Panels B and B′ are surface representation with volume occupied by the domain. C and C′ show the combination of panels A/A′ and B/B′. The SBD structure was obtained after one-to-one threading process using the dvpra1a.pdb structure against the 417–503 sequence of Sip1. The overall structure of the SBD shows the presence of several α-helixes bundled and coiled-coil regions in a closed conformation. The (QxVx)_2_ sequence (as part of aa459–472; red/yellow α-helix panels A and A′) is on the solvent-exposed surface and two polar chains protrude from this α-helix, suggesting a potential role as mediator of the interaction with the hydrophobic corridor in the Smad MH2 domain. In blue: the previously defined SBD aa437–487; red: aa459–472 (with the (QxVx)_2_ sequence); yellow: Q461, V463, Q465 and V467 residues (see main text for further details). X,Y and Z axes are depicted showing the orientation of the two sets of panels.

*In silico* alignment of the Sip1 SBD sequence from different species indicated that the identified 14 aa-long stretch, which corresponds to mouse Sip1 aa459–472 and is identical to aptamer A6 (Figure 1A), has a high level of conservation score within the 51 aa-long SBDs. To identify potential structures and candidate residues that are crucial for and/or mediate Sip1-Smad interaction, we applied 3D modelling, leading to a structural representation (see Figure 2) that however remains hypothetical and needs future experimental confirmation. The mouse Sip1 SBD sequence (as aa437–487) was submitted to the Phyre2 bioinformatics server [47]. This yielded several, but related top score potential secondary and tertiary structures. Subsequent analysis of these with PyMol software suggested a common structure (d1vpra.pdb, see Figure S1A) based on this long SBD sequence. This d1vpra.pdb model was used as template in a one-to-one threading process [47] against the longer aa417–503 segment of mouse Sip1. This approach revealed that an α-helix encompassing the aa459–472 stretch is the central structural element of the SBD (Figure 2A, A′). This element, and in particular the minimal, shorter SBD stretch identified in this study, contains specific residues that may mediate protein-protein interaction. Q461 and Q465 protrude from the α-helix, as shown by surface representation (Figure 2B, B′). Furthermore, this α-helix could according to our preliminary analyses (Leslie, Conidi and Huylebroeck, *unpublished results*) fit well in the hydrophobic corridor present in the Smad3 MH2 domain that is formed by its triple helix bundle and β-strand (Figure S1B) where important residues for the interaction with Sip1 and other SIPs have been identified [54]. This domain and corridor are accessible in activated Smads, wherein the auto-inhibitory interaction between the MH1 and MH2 domain is indeed dissolved.

**Figure 3:**
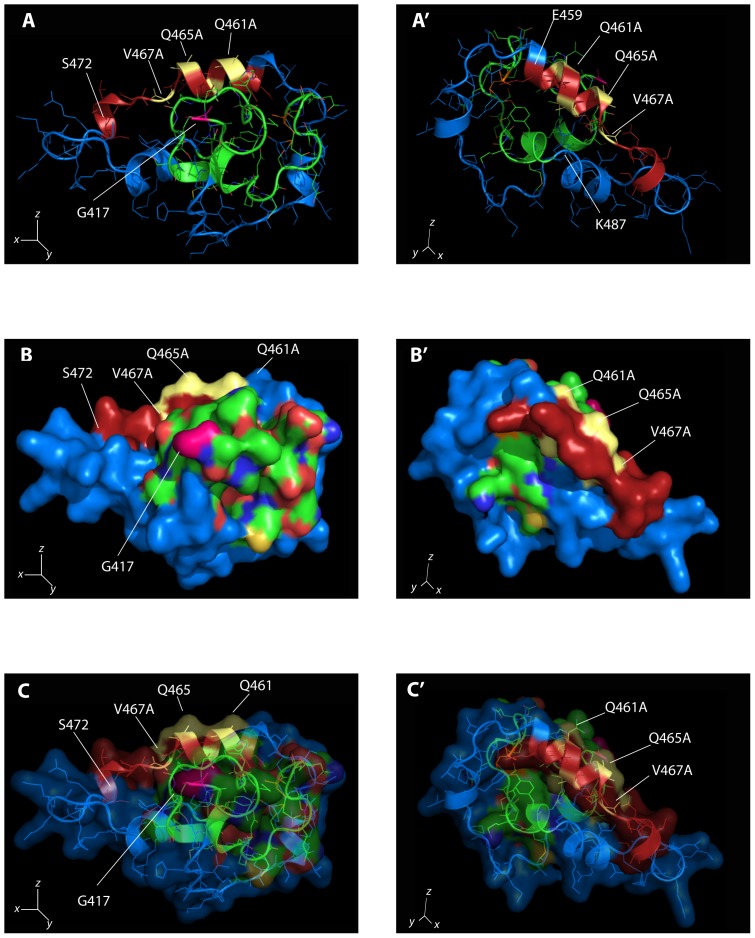
Effect of mutagenesis of specific residues within the Sip1 SBD. Again, two orientations of the same model are depicted (see Figure 2): panels A and A′ represent the “cartoon model” highlighting the structural elements present in the Sip1 SBD; panels B and B′ are surface representation with volume occupied by the domain; C and C′ show the combination of panels A/A′ and B/B′. X,Y and Z axes are depicted showing the orientation of the two sets of panels. No alteration of the overall structural model is seen (compared to the structure presented in Figure 2), but the mutation significantly impairs the candidate contact surface with the Smad MH2 domain.

**Figure 4:**
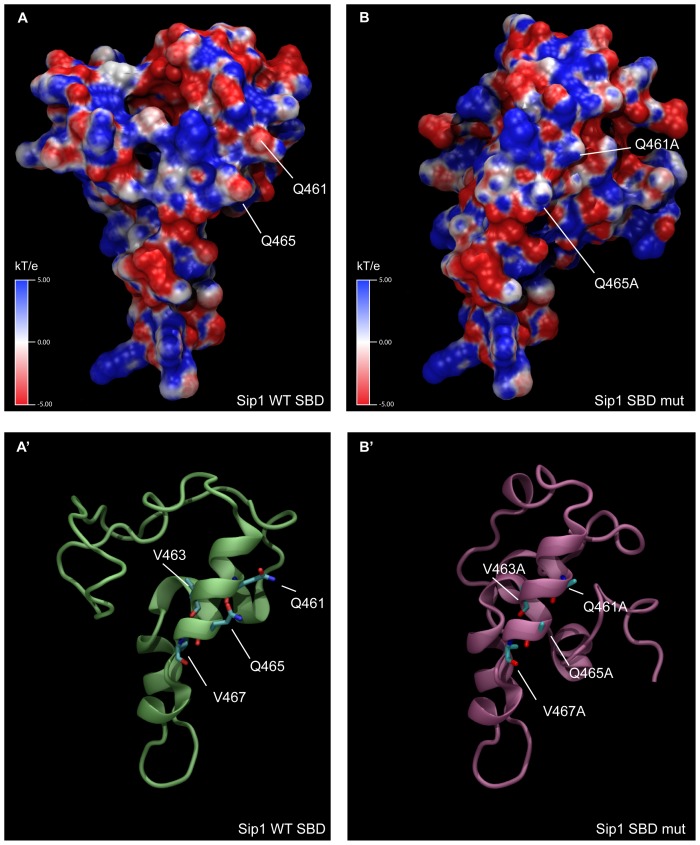
Electrostatic properties of the predicted models for the SBD of Sip1 WT (panel A) and mutant (panel B). Potential isocontours are displayed across a range of −5 to +5 kT/e, depicting negative potential areas (in red). Cartoon representations of the models are also shown, with key residues indicated in stick representation (panels A′ and B′).

**Figure 5:**
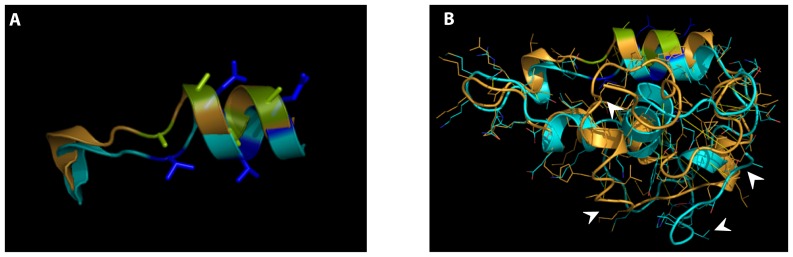
Overlay of Sip1 WT (cyan) and (AxAx)_2_ mutant (ochre) aa459–472 (panel A), highlighting the absence of polar chains protruding from the solvent-exposed surface, and the global SBD domains (panel B). In A blue: WT SBD residues; lime: SBD residues that are mutated to A. Arrowheads indicate highly disordered regions of the two superimposed structures.

Q461 and Q465 are part of a dual repeat of 4 amino acids with Q and V in position 1 and 3, which is highly conserved in Sip1 from different vertebrates (Figure 1A). We refer to this 8aa-long sequence as the (QxVx)_2_ motif in Sip1 and its SBD (in red in Figure 2). We then collectively mutated the amino acids Q461, V463, Q465, V467 to Alanine in a full-length mouse Sip1 backbone (hereafter named Sip1(AxAx)_2_ mutant). These substitutions would ideally affect Sip1-Smad interaction only, *i.e.* without disrupting the predicted structure and folding of this segment in Sip1. To confirm this, we submitted the sequence of such mutant SBD to the Phyre2 server for a one-to-one threading modelling and enquired for its fit with the previously obtained predicted structure. As shown in Figure 3(A, A′), the α-helix would be preserved in the mutant SBD, but the anticipated key Q and V residues would no longer protrude from the surface (Figure 3B, B′) due to the absence of the polar Q residues in particular. Figure 4 shows the electrostatic properties of the predicted WT and mutant (AxAx)_2_ SBD: the negative charges (red areas) in the region of the Q461 to Q465 of WT Sip1 are lost in the mutant. When we superimpose the WT and the SBD mutant structures we obtained a significant root mean square deviation (RMSD), a commonly used measure of dissimilarity or error in protein structure, for the α-helices of the aa459–472 region (RMSD = 0.12) (Figure 5A). The randomness of the *in silico* predicted models, however, infers on the general RMSD value, as also confirmed by the disordered regions shown in Figure 5B (arrowheads).

### Full-length Sip1(AxAx)_2_ Mutant Protein Fails to Bind to Activated Smad Proteins and Still Binds to its DNA Target Sequence

**Figure 6:**
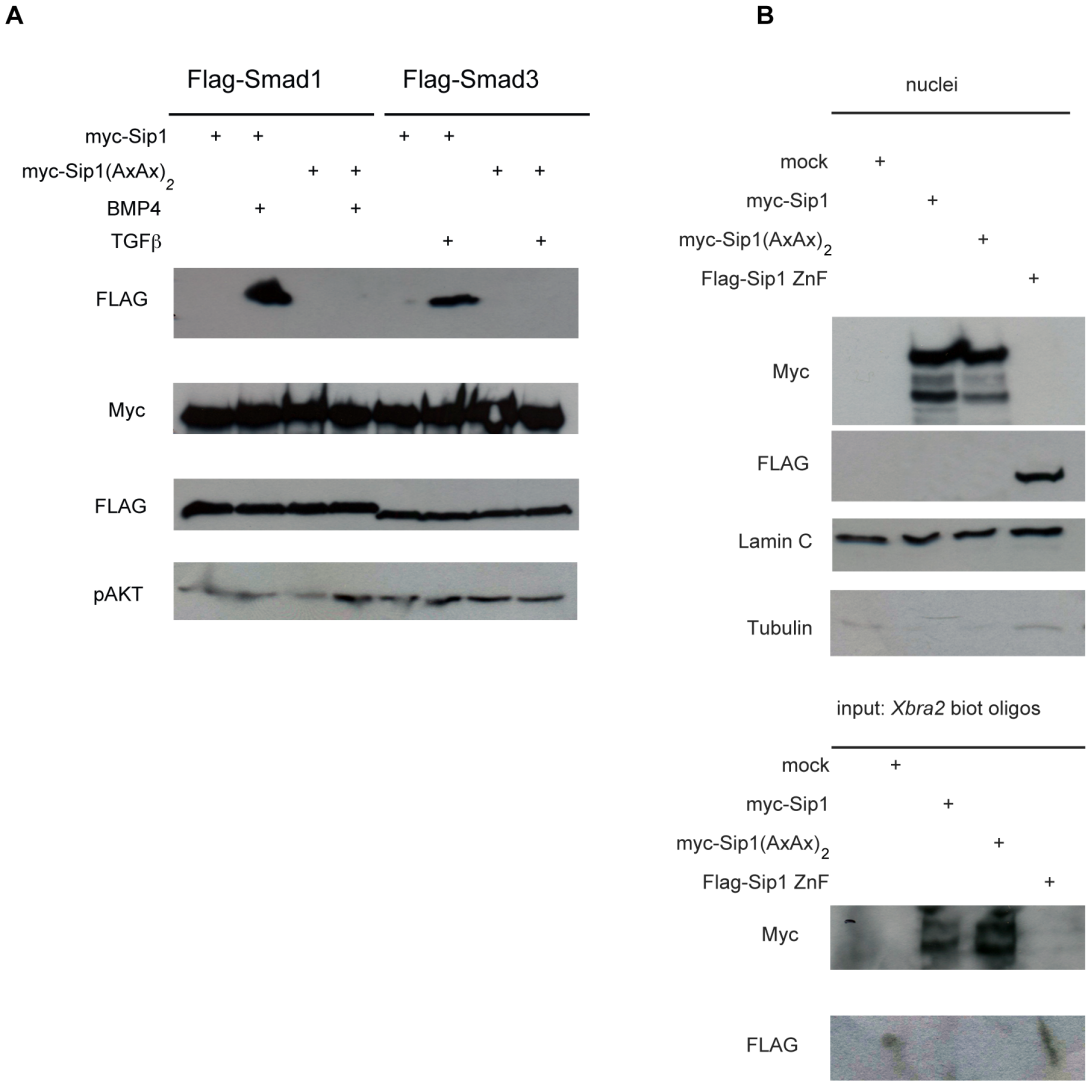
Full-length Sip1(AxAx)_2_ mutant no longer binds to activated Smads, while its DNA-binding property is preserved. A) Site-specific mutagenesis of the QxVx repeats (see Results) in the SBD of full-length (Myc-tagged) Sip1 causes loss of Smad interaction in ligand-activated cells (shown are Smad1 and Smad3). B) Sip1(AxAx)_2_ protein is predominantly nuclear, like WT Sip1. Lamin C was used as marker for the nuclear fraction and Tubulin serves as control to verify the presence of cytoplasmic contamination in the lysates. C) DNA-IP using a segment encompassing the Sip1-binding, E-box containing segment of the *Xbra2* promoter demonstrated that Sip1(AxAx)_2_ is still able to bind to its cognate DNA target sequence [13].

We first tested if the Sip1(AxAx)_2_ mutant binds to Smads. Co-IP experiments in extracts of transfected ligand-stimulated HEK293T cells demonstrated that the interaction between full-length (N-terminally Myc-tagged) Sip1(AxAx)_2_ and activated (Flag-tagged) BMP-Smads and TGFβ-Smads was lost (Figure 6A; Smad1 and Smad3 are shown here, respectively; pAkt detection is included here to confirm pathway activation). In order to verify that the inserted mutations affect Smad binding while keeping other domains functional, in particular the function of each of the two zinc finger clusters in DNA binding, we performed a DNA pull-down experiment (see also [14]), using biotinylated double-stranded deoxyoligonucleotides containing an acknowledged Sip1-binding DNA target sequence. The promoter region of *Xbra2* confers responsiveness to Activin; it contains a cognate Sip1 binding site that is necessary and sufficient, and needs to be intact, in the early *Xenopus* embryo for the proper and brief spatial-temporal control of *Xbra2* [53], just prior to the segregation of the *Xbra* and *Sip1* expression domains into mesoderm and neuroectoderm, respectively [55]–[57]. We selected a 153 bp-long sequence of the *Xbra2* gene promoter (see Materials and Methods), wherein the half sites for DNA binding by Sip1 reside, and where these are separated by 24 bp [13], [55]. Nuclear/cytoplasmic fractionation was performed on HEK293T cells transfected with (Myc-tagged) Sip1 WT [15] or Sip1 (AxAx)_2_ (this study) or with (Flag-tagged) Sip1 ZnF mutant [13] encoding constructs (Figure 6B). We then used the nuclear lysates in the DNA pull-down experiment. Sip1(AxAx)_2_ still bound to the E-box containing *Xbra2* promoter fragment, like Sip1 WT (Figure 6C); the Sip1 ZnF mutant of Sip1 did not (see also [15], tested in *E-cadherin* regulation in MDCK epithelial cells), similar to what has been observed using gel-based electromobility shift analysis [13].

### Sip1 SBD Mutant, Unlike Sip1 WT, does not Display Dose-dependent Down Regulation of TGFβ and BMP-induced, Smad-dependent Reporter Gene Response

**Figure 7:**
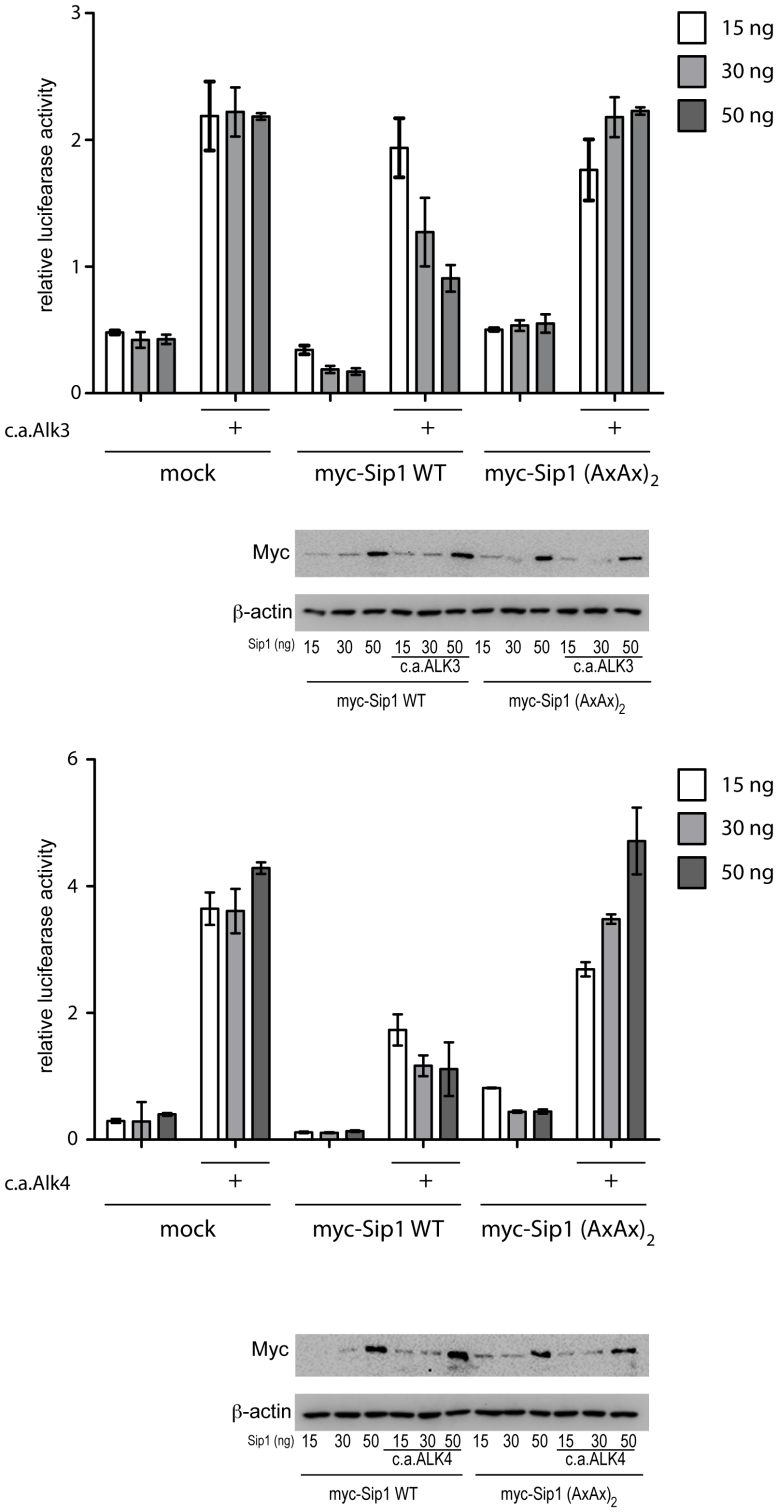
BMP/TGFβ-induced activation of SBE_4_-based luciferase based-promoter is maintained in cells overexpressing the (AxAx)_2_ mutant. HEK293T cells were transfected with WT or SBD mutant Sip1 encoding plasmids in presence (+) or absence of a c.a.Alk3 (panel A) or c.a.Alk4 (panel B) receptor. Increasing concentrations of Sip1 WT plasmid result in a down regulation of BMP or TGFβ induced gene response, effect that is attenuated by co-transfection of the c.a.Alk3/Alk4 encoding plasmids. On the other hand, the Smad binding mutant Sip1(AxAx)_2_ is no longer able to repress such gene response. Blots show the expression levels of the Sip1 (WT and mutant) encoding plasmids transfected. Images were acquired using a Digital Chemiluminescence System (Bio-Rad).

To evaluate whether the Sip1 SBD mutant Sip1(AxAx)_2_ is able to interfere with Smad-mediated gene response induced by TGFβ or BMP we performed a promoter-reporter assay based on a luciferase construct containing a repeat of the SBE (*i.e.* SBE_4_) of the Pai1 promoter. This construct is responsive to both TGFβ and BMP [58]. HEK293T cells were transfected with increasing concentration of Sip1 WT or Sip1(AxAx)_2_ mutant encoding plasmids together with plasmids encoding for constitutive active forms of Alk3 or Alk4 receptors to activate the BMP or Nodal/Activin Smad pathway, respectively (Figure 7). BMP pathway activation showed a 5-fold increase in induction of the SBE_4_-based promoter in control cells (mock vs. mock+c.a.Alk3). Sip1-WT transfected cells showed a dose-dependent reduction in BMP-Smad mediated promoter activation, while the Sip1 SBD mutant was not and in fact restored the BMP-dependent induction of the SBE_4_-based promoter (Figure 7A). The same effect was observed in cells in which Smad2 and 3 were activated by co-transfection with c.a.Alk4 (Figure 7B). Hence, both the TGFβ and BMP responses recover when cells were transfected with the Sip1 SBD mutant. Therefore we can conclude that Smad signaling in Sip1-SBD mutant expressing cells and that DNA binding of the Sip1 SBD mutant are still effective.

### Sip1-Smad Interaction in Ligand-activated Cells, as Probed by Inclusion of the Sip1(AxAx)_2_ Mutant, Suggests a Role in Transcriptional Repression of *Cdh1* in Epithelial Cells

We and others have shown that Smad3 and Sip1 join a longer list of transcriptional regulators (*e.g.* Id proteins, Snail, Slug, and the Sip1-related δEF1/Zeb1) that play a crucial role in downregulating and repressing the expression of key epithelial specific proteins (*e.g.* E-cadherin, Cdh1) while upregulating mesenchymal marker genes in EMT [17], [46], [59]–[63]. We therefore tested first whether our aptamers could interfere with TGFβ-induced EMT in the NMe cell line [14]. However, we did not observe an inhibition of EMT in aptamer-producing NMe cell cultures treated with TGFβ1 for 48 hours (*data not shown*). This could be due to the high levels of endogenous Sip1 induced by TGFβ in these cells (as documented in [14]) in combination with the low efficiency of transfection with the aptamer-encoding vectors in NMe cells.

**Figure 8:**
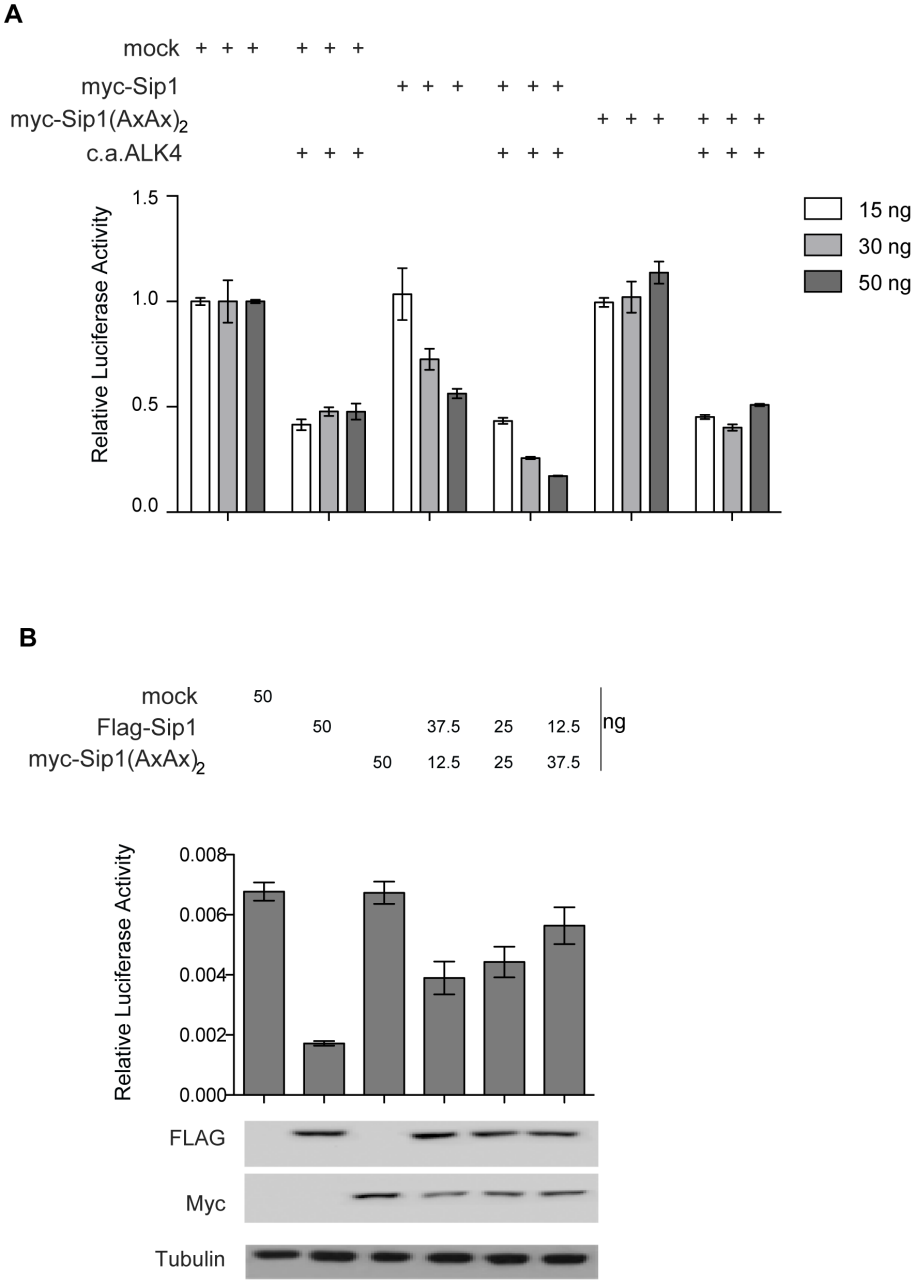
Sip1(AxAx)_2_ represses *Cdh1* promoter based reporter activity in a c.a.Alk4/activated Smad-independent and dominant-negative, DNA-binding fashion. A) Luciferase reporter assay showing the inhibition of the transcriptional activity of the *Cdh1* promoter mediated by WT Sip1 (as already described in several studies). Upon transfection of the cells with a plasmid encoding the Sip1(AxAx)_2_ mutant, no repressive effect was observed on the co-transfected *Cdh1* promoter in the absence of c.a.ALK4. In addition, the WT Sip1 dose-dependent repression in the presence of c.a.ALK4 is lost upon co-transfection of the Sip1(AxAx)_2_ mutant. B) Competition assay in cells co-transfected with WT Sip1 (human; Flag-tagged) and mutant Sip1(AxAx)_2_ encoding plasmids at different ratios (total amount of transfected DNA in all cases is 50 ng). Restoration of the levels of *Cdh1* promoter activity is seen in the presence of the Sip1(AxAx)_2_ SBD mutant, which acts as a DNA-binding dominant-negative Sip1 (in terms of Smad interaction), suggesting that the transcriptional repression mediated by Sip1 is strictly Smad-dependent and requires Smads bound as co-factors. Error bars represent standard deviations.

Repression of the endogenous *Cdh1* promoter or a transiently transfected *Cdh1* promoter based reporter is frequently used as functional read-out for Sip1 activity. Here, we checked whether Sip1(AxAx)_2_ is still able to mediate transcriptional repression of *Cdh1* promoter driven luciferase. Cells were transfected with Sip1 WT or Sip1(AxAx)_2_ encoding vectors together with a vector encoding for c.a.Alk4. The levels of the *Cdh1* promoter-driven reporter transcript were down regulated by increasing amounts of Sip1 WT vector, and the Sip1 dose-dependent repression was stronger upon co-transfection with c.a.Alk4 (Figure 8A). The Sip1(AxAx)_2_ encoding vector was not able to repress *Cdh1* in a dose-dependent manner (Figure 8A). Co-transfection of both Sip1 plasmids (Figure 8B) at different ratios shows that Sip1(AxAx)_2_ inhibited the effect of Sip1 WT already at a 3∶1 ratio of Sip1 WT versus Sip1(AxAx)_2_. Altogether, we conclude that Sip1(AxAx)_2_ works as a dominant-negative Sip1, operationally defined here as a transcription factor still binding to DNA and at the same time being insensitive to (c.a.Alk4) activated Smad in NMe cells.

**Figure 9:**
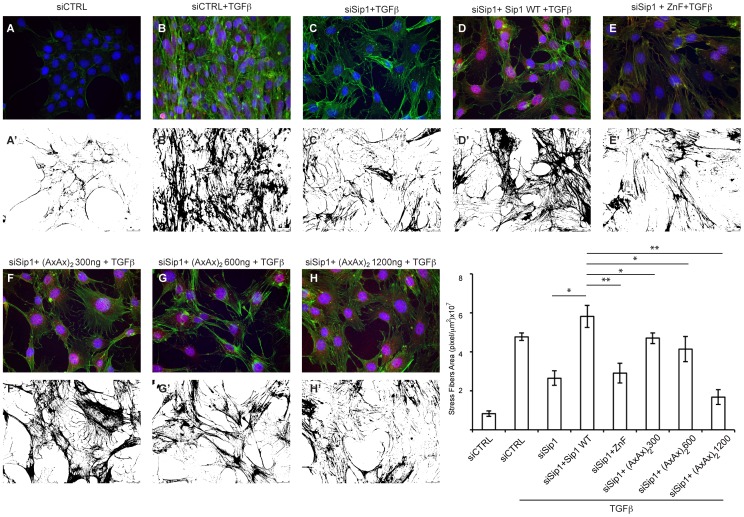
Smad-Sip1 interaction is active during TGFβ-induced EMT in NMe cells. Reduction of endogenous Sip1/Zfhx1b levels using specific siRNAs (A–C) and rescue using WT Sip1 (D). Different concentrations of Sip1(AxAx)_2_ (E–G) or ZnF (F) mutant encoding plasmids do not show a marked inhibition of the EMT process in TGFβ1-stimulated cells. Only a significant reduction in the formation of actin stress fibers (graph in panel I) could be observed with increasing concentration of the Sip1(AxAx)_2_ mutant. Blue DAPI; green: F-actin; red: anti-Sip1 (panels A–C), anti-myc (panels D–G), anti-Flag (panel H). Panels A′ to H′ show the stress fibers images quantified (see Materials and Methods for details) in panel H. *p<0.05; **p<0.01, Student t-test.

Next, we overexpressed full-length Sip1(AxAx)_2_ in NMe cells undergoing EMT [14], [46], while knocking down the endogenous levels of Sip1. For this purpose, we co-transfected the cells with siRNA for mouse Sip1 and 1200 ng/ml Sip1 WT vector or increasing concentrations of Sip1(AxAx)_2_ vector (*i.e.* 300, 600 and up to 1200 ng/ml), and then stimulated the cells with TGFβ for 48 hours. As shown in Figure 9, cells transfected with increasing amounts of Sip1(AxAx)_2_ encoding vector showed less stress fiber formation (1.6, 1.8 and 3.6 times less compared to the control; for quantification, see Materials and Methods), even though the overall morphology of the cells was not affected. The failure of Sip1(AxAx)_2_ to down regulate endogenous *Cdh1* transcription and the observed reduced formation of stress fibers, a hallmark of EMT, suggest that activated Smads have at least to co-operate with Sip1 in the repression of certain key target genes in EMT. In the case of the EMT gene *Cdh1* it remains unclear as to whether the Smad-Sip1 co-operation is the result of a direct interaction between Sip1 and Smads, or not.

### Probing Sip1-Smad Interaction Dependency with SIP1(AxAx)_2_ *ex vivo*

We recently discovered that Sip1 controls *in vivo* (using conditional knockout mice) and *ex vivo* (using forebrain slices from mice, including from our knockout mice) the directed tangential migration of cortical GABAergic interneurons from the medial ganglionic eminences of the ventral part of the telencephalon to the cortex [38]. To achieve this directed migration, interneurons use a huge array of molecular mechanisms including motogenic factors, guidance cues and transcription factors, including Sip1, which is a key transcription factor for the guided migration of these interneurons to the cortex [38]. Interestingly, Smad signaling might co-regulate this guidance process as well because several TGFβ/BMP pathway components are expressed in the ventral telencephalon. In addition, Smads have been suggested to function in interneuron migration [64].

**Figure 10:**
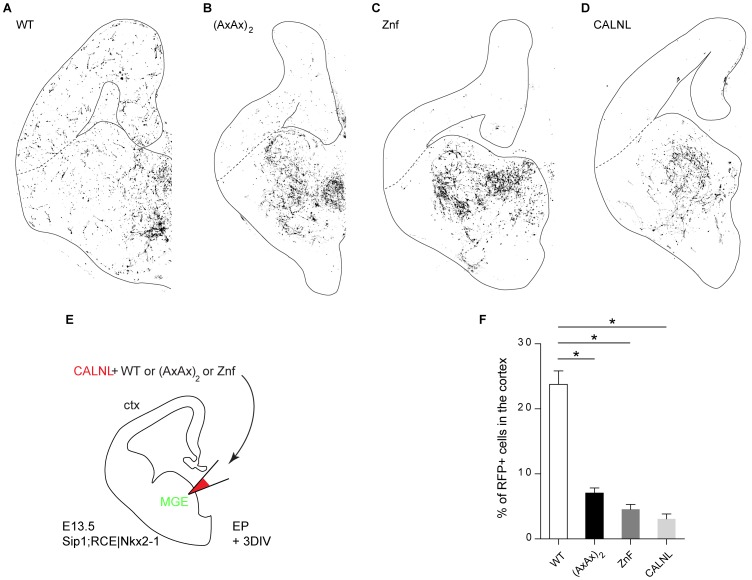
GABAergic interneuron migration to the cortex is mediated by Sip1 in a Smad-dependent way. The focal electroporation experiment is schematically represented in panel E (for more details, see Materials & Methods, and see van den Berghe *et al.*, 2013). Sip1 WT or Sip1 SBD domain mutant construct (AxAx)_2_, and ZnF, were co-electroporated with a conditional dsRed-encoding plasmid (CALNL) to mark targeted cells in Sip1;RCE|Nkx2-1 brain slices (E13.5). After 3 days *in vitro* (DIV), only 3.10% ±0.73% (n = 9 slices) of the *Sip1* KO interneurons in the control condition (CALNL only) is able to reach the cortex compared to 23.77% ±2.06% (n = 28 slices) when a Sip1 WT construct is electroporated. The Sip1 domain mutants are not able to rescue the interneuron migration, Sip1(AxAx)_2_ mutant: 7.06±0.77% (n = 29 slices) and Sip1 ZnF mutant: 4.55% ±0.75% (n = 18 slices). Quantification is shown in panel F. Error bars represent the SEM of 2 independent experiments, *p<0.0001, Chi-square test.

We wondered whether the directed migration of GABAergic interneurons by Sip1 depends on the interaction of Sip1 with Smads. In particular, we investigated whether focal electroporation (see also Materials and Methods section, and [53]) of an expression vector for Sip1(AxAx)_2_ could rescue the migration deficit observed in *Sip1* knockout brain slices. Vectors encoding Sip1 WT, Sip1(AxAx)_2_, the Sip1 ZnF mutant, respectively, each together with the reporter plasmid CALNL, and the reporter plasmid on itself, were focally electroporated in organotypic brain slices of E13.5 Sip1;RCE|Nkx2-1 mutant embryos (for a representation of the experimental set-up, see Figure 10E). In the latter embryos/brains, *Sip1* was deleted in MGE-derived interneurons resulting in disruption of their migration to the cortex [38]. First, we determined which concentration of Sip1 WT vector resulted in the most efficient rescue of the migration-arrest of the interneurons to the *Sip1* knockout cortex. Therefore, we compared the Sip1 WT constructs at a final concentration of 1 µg/ µl, 0.5 µg/µl or 0.1 µg/ µl (mixed with the CALNL plasmid at 1 µg/ µl) versus the control plasmid (CALNL only, at 1 µg/ µl). For each condition, we quantified the total number of RFP-positive (RFP+, *i.e.* red) cells in the slice and calculated the percentage of RFP+ neurons that could reach the cortex after 3 days of culture. Electroporation of Sip1 WT vector at a final concentration of 0.5 µg/ µl resulted in the best rescue, *i.e.* the highest fraction (in %) of RFP+ neurons in the cortex (*data not shown*).

All Sip1 constructs were then used at the concentration of 0.5 µg/ µl in rescue experiments. Restoring Sip1 levels with the electroporated Sip1 WT vector resulted in a clear rescue of the interneuron migration/location defect (23.77%, n = 28 slices) compared to the negative control CALNL (3.10%, n = 9 slices, p<0.0001) (Figure 10A,D and quantification in F; see also [38]). However, the Sip1 mutant constructs (AxAx)_2_ and ZnF, respectively, failed to rescue (7.06%, n = 29, p<0.0001 and 4.55%, n = 18 slices, p<0.0001 respectively) (Figure 10B,C,F). These data indicate for the first time that the SBD of Sip1 needs to be intact and is mandatory for a Sip1-dependent process *in vivo*, *i.e.* normal interneuron migration in the embryonic forebrain.

## Discussion

We mapped the critical residues for Smad interaction in the Smad-interacting protein Sip1, which as DNA-binding transcription factor is intensively studied in embryogenesis and tumorigenesis. Using a peptide aptamer approach along with structural modelling we identified an important predicted α-helical structural element of its SBD and redefine the 51 aa-long SBD (see [12]) as a 14 aa-long stretch. To our knowledge this is one of the rare descriptions of an SBD for which constrained peptides have been used to map specific residues mediating interaction between Smad and a quite large SIP transcription factor. We also found that the binding of the two classes of Smad (2/3 and 1/5/8) depend on the same sequences within this short SBD. Furthermore, collective mutation of four critical residues in the QxVx repeat of this short SBD is sufficient to abolish Sip1-Smad interaction, while not affecting the DNA-binding property of such full-length, mutant Sip1. The identified (QxVx)_2_ motif in the Sip1 SBD is not present in any other SIP SBD, including the previously proposed SBD PNx5ahx3IPPh (where a is an acidic residue, h is an hydrophobic residue, and x is any residue [65].

The binding with both TGFβ and BMP activated Smads is dependent on the same key residues in the Sip1SBD. Hence, based on these results, it will be impossible to design and test, including *in vivo* and *ex vivo*, variants of Sip1 that would bind selectively to one of the two classes of Smad only, which would have been very interesting tools. This means also that using the Sip1(AxAx)_2_ protein it will be difficult to test if and how, in cells exposed to both TGFβ and BMP signals, Sip1 achieves the appropriate eventual response under this co-stimulation condition. The putative role of Sip1 in balancing TGFβ versus BMP Smad signaling may also be determined by additional mechanisms, including the respective receptor levels, Smad partner choice by other SIPs within these cells or - more upstream in the signal transduction pathway - Smad (out)competition (as recently reported for dual-active Activin receptors in *Drosophila*; [66]). The eventual global response, including the dominant Smad choice for Sip1, may therefore be cell type/context dependent. Such other factors might be the level of the expressed and/or activated Smad partners in the first place, but also the involvement of other Sip1 domains (*e.g.* the NIM in Sip1 [22]), the post-transcriptional modification of Sip1 and/or Smads, or feedback mechanisms that operate in these events.

Peptide aptamers have been used to interfere with TGFβ signaling components such as FoxH1, Lef1, CBP and SARA [44], [45]. SARA-based Smad-binding aptamers and their expression in NMuMG epithelial cells resulted in a block of TGFβ-induced EMT and an inhibition of Smad3 activity [45]. When we transfected our aptamers in the NMe cell line, which is a NMuMG-derived cell line producing stably and homogeneously throughout the cell cultures high levels of E-cadherin, and then stimulated with TGFβ1 for 48 hours, we could not observe a significant block of the EMT process. The overall levels of endogenous E-cadherin (Cdh1) in the cultured cells persisted, and in addition their Smad response was not affected (*data not shown*). We suggest this result is due to the low efficiency of transfection in Nme cells in the first place. Another likely cause is the previously documented induction of the endogenous *Sip1* gene following TGFβ1 treatment of and hence EMT induction in NMe cells in the same timeframe as the one used by us here [14]. SARA-derived aptamers may successfully impair EMT (as reported in [45]) because in this case a unique SIP and upstream (cytoplasmic) effector is targeted whose activity is mainly to present TGFβ-Smad to the receptor complex. Doing so, SARA promotes Smad phosphorylation by the type I receptors at the early endosome level [67], which amplifies the signaling. On the other hand, activation of the TGFβ pathway in epithelial cells can lead to expression of other transcription factor encoding genes than *Sip1*, *i.e.* those encoding bHLH factors (like Twist), Snail/Slug, and the Sip1-related, non-Smad binding protein δEF1 (also named Zeb1 and Zfhx1a), suggesting co-operation and likely an hierarchy in EMT regulation. Interestingly, Snail also binds to the Smad3/4 complex [68], making a similar structural analysis of its SBD as the one done here for Sip1 and their co-operation in *Cdh1* regulation very interesting, and both *Snail* and *Sip1* gene transcription are also regulated by Smad signaling. In addition, it has been shown that re-expression of epithelial markers as well as re-establishment of epithelial morphology in NMuMG cells that have undergone EMT occur only when both Sip1 and the related δEF1 protein, and the Rho pathway, are inhibited [17]. Others and we have already shown that Smad3 is necessary for TGFβ-induced EMT [46], [59]. In our case, targeting Sip1-Smad2/3 interaction in EMT in the NMe cell line, even when we would be able to achieve high efficiency via transfection (or viral transduction), would then not affect the activity of some of these other transcription factors and hence abrogate neither EMT nor TGFβ-promoted tumorigenesis.

The connection between Sip1 binding and activated Smad binding to the *Cdh1* regulatory region has thus far not been directly shown. Furthermore, the Sip1/Zeb2-related protein δEF1/Zeb1a also downregulates *Cdh1*, but does not detectably bind to Smads (our *unpublished data*), suggesting that - at least in some epithelial cells - the Zeb family members down regulate *Cdh1* in a Smad-independent manner after TGFβ-Smad mediated EMT has been induced at the level of other target genes for Smad. In line with this, Sip1 and δEF1 have been found to be necessary, but not sufficient, for TGFβ-induced EMT in NMuMG cells, requesting the upstream action of the TGFβ-induced transcription factor encoding gene *Ets-1* [69]. A Smad-binding motif is however present in *Cdh1* at +46, still in the untranslated region of the gene. This sequence is highly conserved both in human and mouse. Whether Sip1 and Smad are simultaneously bound to these *Cdh1* regions and – if they form a complex here – who influences who in such complex is still an intriguing question. We have previously published evidence - in the process of myelination – that BMP-Smad activated genes become down regulated/repressed in the presence of and in conjunction with Sip1 [39]. However, we find here – using the Sip1 SBD mutant constructed in this study – for the first time that an intact SBD is needed in Sip1 to down regulate endogenous *Cdh1* transcription in conditions of TGFβ pathway activation (using c.a.Alk4).

The multi-functionality and versatility, including interaction with many SIPs, of Smad proteins are impressive. Indeed, tens of different SIPs bind to Smads via very diverse Smad-binding primary sequences. Interestingly, the majority of the SIPs for which a similar study has been done as ours here, share in their SBD the same structural element, *i.e.* an α-helix that is crucial for binding of the SIP to Smad. Our 3D modelling of the Sip1 SBD reveals that this short domain may fold on itself, forming a hinge-like structure, and that the exposed part is α-helical. The key residues in this short α-helix seem to play a role as gatekeeper, as they protrude from the surface. Schiro and co-workers [54] have also reported that in the MH2 domain of Smad3 the residue Y298 is crucial for binding with Sip1, and that mutation of Y226, V356 and W406, resulted in reduced binding of Smad3 to SARA, Ski and Sip1 SBDs. These residues reside in the hydrophobic corridor of the MH2 domain [54]. When we docked *in silico* our predicted model with the MH2 domain of Smad3 (pdb: 1MK2), Q461 and Q465 of Sip1 SBD are suggested to fit in this corridor and be able to mediate the interaction with the MH2 domain (*data not shown*). For this reason, we mutated these residues within full-length Sip1 and tested whether we could interfere with Sip1-Smad interaction and at the same time preserve the functionality of the other domains of Sip1, in particular the DNA-binding. The results confirmed the essential role of these residues for mediating binding of Sip1 with activated Smads. We also conclude that the Sip1-SBD mutant Sip1(AxAx)_2_ no longer binds to type I receptor (Alk4) activated Smad(s). It works as a dominant-negative in this respect, but is still a DNA-binding Sip1.

Recently we described a key-role for Sip1 during migration of GABAergic interneurons to the cortex during mouse brain development [38]. These neurons originate mainly in the MGE of the embryonic ventral telencephalon and follow precise paths to the cortex, subject to attractive cues and repulsive cues (including Unc5b-mediated ones, see [38]), where they establish essential inhibitory connections with excitatory projection neurons. This phenotype can be rescued both *in vivo* by conditional, MGE-specific Sip1 transgene expression and *ex vivo* by focal electroporation of Sip1 WT vectors in embryonic forebrain slices ([38: van den Berghe, Conidi, Seuntjens and Huylebroeck, *unpublished results*). Importantly, the Smad-binding deficient Sip1(AxAx)_2_ mutant could not rescue interneuron migration to the cortex. We therefore conclude that the crucial role of Sip1 in precise interneuron migration and cue interpretation is dependent on the presence of and interaction of Sip1 with activated Smads. Further *in vivo* and *ex vivo* work in the interneuron field is needed to document whether modulation of essential genes along the entire migratory route, or a part of this trajectory, of cortical interneuron is subject to TGFβ signaling via Smads in general and the SBD of Sip1 in particular. Both TGFβ and BMP signaling may take place in this brain region: indeed, focal electroporation of dominant negative forms of Smad1, Smad2 and Smad4 resulted in a decreased cell migration to the cortex [64].

Taken together, our study identifies within the intensively studied SIP-TF Sip1 a structural element and short sequence that mediates Sip1-Smad interaction. The subtle Sip1(AxAx)_2_ SBD mutant constructed here still binds to its cognate target DNA sequences, but no longer binds to Smad proteins. This Sip1 mutant provides an important new tool to identify Smad-(in)dependent processes as well as target genes resulting from obligatory Sip1-Smad interaction, either in cell culture, *ex vivo* or in a conditional knock-in mouse model. Approaches that involve screening of co-expressed cDNAs or miRNAs, or of natural or synthetic compounds, which interfere with Sip1-Smad interaction in cultured cells, would also benefit from inclusion of this mutant in these experiments.

## Supporting Information

Figure S1 **Initial model of the Sip1 SBD obtained by homology prediction and crucial residues for Sip1 interaction on MH2 domain of Smad3.** A) dvpra1a.pdb representation. The 459–472 region is part of a longer α-helix. B) Residues (depicted in orange) of the MH2 domain of Smad3 (pdb: 1MK2) important for interaction with Sip1 and other SIPs according Schiro et al. (2012) [54].
(TIF)
