# Supplementary figures and images for: Four Amino Acids within a Tandem QxVx Repeat in a Predicted Extended α-Helix of the Smad-Binding Domain of Sip1 Are Necessary for Binding to Activated Smad Proteins
Source: PLoS One. 2013 Oct 11;8(10):e76733. doi: 10.1371/journal.pone.0076733 (PMC3795639; doi:10.1371/journal.pone.0076733)

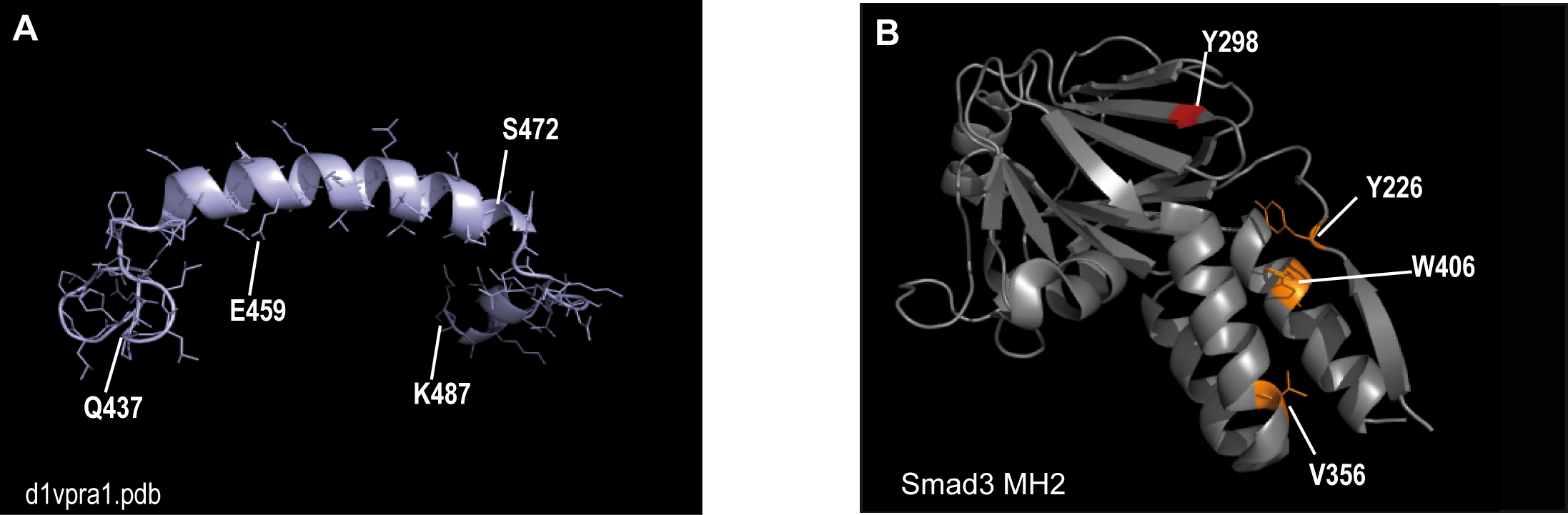

Supplement: Figure S1 — Initial model of the Sip1 SBD obtained by homology prediction and crucial residues for Sip1 interaction on MH2 domain of Smad3. A) dvpra1a.pdb representation. The 459–472 region is part of a longer α-helix. B) Residues (depicted in orange) of the MH2 domain of Smad3 (pdb: 1MK2) important for interaction with Sip1 and other SIPs according Schiro et al. (2012) [54]. (TIF) [file pone.0076733.s001.tif]
